# PPARδ Activation Mitigates 6-OHDA-Induced Neuronal Damage by Regulating Intracellular Iron Levels

**DOI:** 10.3390/antiox11050810

**Published:** 2022-04-21

**Authors:** Won Jin Lee, Hyuk Gyoon Lee, Jinwoo Hur, Gyeong Hee Lee, Jun Pil Won, Eunsu Kim, Jung Seok Hwang, Han Geuk Seo

**Affiliations:** College of Sang-Huh Life Sciences, Konkuk University, 120 Neungdong-ro, Gwangjin-gu, Seoul 05029, Korea; windfall@konkuk.ac.kr (W.J.L.); krci-12@daum.net (H.G.L.); wlsdn91@konkuk.ac.kr (J.H.); kyung9642@daum.net (G.H.L.); wjp0505@konkuk.ac.kr (J.P.W.); np-gennao@hanmail.net (E.K.); mathking83@hanmail.net (J.S.H.)

**Keywords:** ferroptosis, 6-hydroxydopamine, iron homeostasis, iron regulatory protein 1, peroxisome proliferator-activated receptor δ

## Abstract

Intracellular iron accumulation in dopaminergic neurons contributes to neuronal cell death in progressive neurodegenerative disorders such as Parkinson’s disease. However, the mechanisms of iron homeostasis in this context remain incompletely understood. In the present study, we assessed the role of the nuclear receptor peroxisome proliferator-activated receptor δ (PPARδ) in cellular iron homeostasis. We identified that PPARδ inhibited 6-hydroxydopamine (6-OHDA)-triggered neurotoxicity in SH-SY5Y neuroblastoma cells. PPARδ activation with GW501516, a specific PPARδ agonist, mitigated 6-OHDA-induced neuronal damage. Further, PPARδ activation also suppressed iron accumulation, which contributes to 6-OHDA-induced neuronal damage. PPARδ activation attenuated 6-OHDA-induced neuronal damage in a similar manner to that of the iron chelator deferoxamine. We further elucidated that PPARδ modulated cellular iron homeostasis by regulating expression of divalent metal transporter 1, ferroportin 1, and ferritin, but not transferrin receptor 1, through iron regulatory protein 1 in 6-OHDA-treated cells. Interestingly, PPARδ activation suppressed 6-OHDA-triggered generation of reactive oxygen species and lipid peroxides. The effects of GW501516 were abrogated by shRNA knockdown of PPARδ, indicating that the effects of GW501516 were PPARδ-dependent. Taken together, these findings suggest that PPARδ attenuates 6-OHDA-induced neurotoxicity by preventing intracellular iron accumulation, thereby suppressing iron overload-associated generation of reactive oxygen species and lipid peroxides, key mediators of ferroptotic cell death.

## 1. Introduction

Parkinson’s disease (PD), a progressive neurodegenerative disorder, is defined by loss of dopaminergic neurons in the substantia nigra, accompanied by prominent neuropathological symptoms such as tremor and bradykinesia [1]. Although multiple approaches to PD treatment have been evaluated in experimental and clinical trials, the only clinically available PD therapy is replacement of dopamine (DA) with the DA precursor levodopa [2,3]. Because continuous degeneration of dopaminergic neurons does not allow conversion of the precursor to dopamine in advanced PD [3], developing a therapeutic that can prevent degeneration of dopaminergic neurons would address a substantial unmet clinical need. 6-hydroxydopamine (6-OHDA) is a neurotoxic, hydroxylated form of DA that contributes to the degeneration of both noradrenergic and dopaminergic neurons in PD [4]. 6-OHDA is transported into neurons by binding to cell membrane transporters expressed in both noradrenergic and dopaminergic neurons [5]. 6-OHDA has been reported to induce unilateral lesions in experimental models of PD [4]. Recently, it was reported that 6-OHDA enhances iron concentration in vivo as shown with microdialysis in a recent study [6].

Ferroptosis is a recently recognized form of non-apoptotic cell death and is implicated in the pathogenesis of PD [7,8]. Ferroptosis is initiated by the generation of highly toxic hydroxy radicals via the Fenton reaction of hydrogen peroxide with ferrous iron and is distinct from other mechanisms of cell death, including apoptosis [7,9]. Unlike apoptosis, ferroptosis is characterized by intracellular accumulation of iron and reactive oxygen species (ROS)-induced formation of lipid peroxides [7,10]. Recently, ferroptosis has been implicated in multiple pathological processes in neurological disorders, cardiomyopathy, and ischemia-reperfusion injury [11,12,13].

Cellular iron homeostasis is associated with the pathogenesis of neurodegenerative disorders such as Alzheimer’s disease (AD), Huntington’s disease (HD), and PD, in which ferroptotic signaling compromises neuronal function and promotes cell death [11,14]. Furthermore, intracellular iron levels are increased in the substantia nigra region of PD patients [15,16], and iron homeostasis is disrupted in PD patients and animal models due to abnormal expression of either divalent metal transporter 1 (DMT1), an iron importer, or ferroportin 1 (FPN1), an iron exporter [17,18,19]. Both DMT1 and FPN1 expression are regulated by iron regulatory protein 1 (IRP1), which binds mRNA stem-loop structures known as iron-response elements (IREs) located in the 5′- and 3′-untranslated regions (UTRs) of FPN1 and DMT1 mRNA, respectively [20]. It is known that IRP1 binds 5′-UTR IREs to repress mRNA translation, thus suppressing target gene protein levels [21]. On the contrary, IRP1 binds 3′-UTR IREs to suppress mRNA degradation, increasing target gene protein levels [21]. Thus, modulation of IRP1 expression and function is a potential therapeutic strategy for iron-related neurodegenerative disease such as PD.

Peroxisome proliferator-activated receptor (PPAR) is a nuclear receptor that functions as a ligand-dependent transcription factor, controlling the expression of multiple target genes involved in diverse biological processes [22]. In the brain, PPAR α or γ subtype demonstrated involvement in processes of brain physiology such as ethanol consumption, anti-inflammatory function, motivation and associated learning [23,24,25]. PPARδ is ubiquitously expressed in mammals, and in neuronal tissue its expression is higher than that of the PPAR subtypes PPAR α and γ, suggesting a more prominent role for PPARδ in the central nervous system [26]. A recent study demonstrated that ligand-dependent activation of PPARδ elicits neuroprotective effects against neurotoxicity induced by the neurotoxin 1-methyl-4-phenyl-1,2,3,6-tetrahydropyridine (MPTP) by preventing striatal dopamine depletion [27]. In addition, the PPARδ agonist GW501516 suppressed inflammatory processes to alleviate motor impairment, dopaminergic neurodegeneration, and midbrain dopamine depletion in an MPTP-induced PD mouse model [28]. These results are consistent with our previous report demonstrating that PPARδ activation attenuates glutamate-induced neurotoxicity by modulating oxidative stress and intracellular calcium levels [29]. PPARδ also suppresses glutamate release in lipopolysaccharide-induced neuroinflammation, so in addition to its direct effects against glutamate neurotoxicity, PPARδ could suppress glutamate release in the context of neuroinflammation [30]. 

Although recent studies demonstrated the association of PPARs with ferroptosis [31,32], the role of PPARδ in brain ferroptosis was not fully investigated despite the high expression level in the brain compared to other PPAR subtypes. We identified that activation of PPARδ with GW501516 inhibits 6-OHDA-triggered neuronal damage by regulating intracellular iron homeostasis, which is mediated by downregulation of IRP1 in SH-SY5Y cells treated with 6-OHDA. These results suggest that PPARδ is a potential therapeutic target in ferroptotic neurodegenerative diseases.

## 2. Materials and Methods

### 2.1. Materials

Puromycin (Cat# P8833), deferoxamine mesylate (DFO, Cat#D9533), 6-hydroxydopamine hydrobromide (6-OHDA, Cat# H116), and lentiviral particles expressing non-targeting or PPARδ-targeting shRNA were purchased from Sigma-Aldrich (St. Louis, MO, USA). GW501516 (Cat# ALX-420-032) was purchased from Enzo Life Sciences (Farmingdale, NY, USA). Monoclonal antibodies specific for α-tubulin (1:2000 dilution; Cat# SC-23948, RRID:AB_628410), DMT1 (1:3000 dilution; Cat# SC-166884, RRID:AB_10610255), and IRP1 (1:200 dilution; Cat# SC-166022, RRID:AB_2273699) were purchased from Santa Cruz Biotechnology (Dallas, TX, USA). Monoclonal anti-ferritin (1:1000 dilution; Cat# ab75973, RRID:AB_1310222), anti-GPX4 (1:2000 dilution; Cat# ab125066, RRID:AB_10973901) antibodies and a polyclonal anti-transferrin receptor protein 1 antibody (TfR1, 1:1000 dilution; Cat# ab84036, RRID:AB_10673794) were purchased from Abcam (Cambridge, UK). Polyclonal anti-FPN1 antibody (1:4000 dilution; Cat# NBP1-21502, RRID:AB_1660490) was purchased from Novus Biologicals (Centennial, CO, USA). Monoclonal antibody specific for IRP2 (1:1000 dilution; Cat# 37135, RRID:AB_2799110) was purchased from Cell Signaling Technology (Danvers, MA, USA). HRP-conjugated polyclonal antibodies specific for mouse immunoglobulin G (1:5000 dilution; Cat# GTX213111-01, RRID:AB_10618076) and rabbit immunoglobulin G (1:5000 dilution; Cat# GTX213110-01, RRID:AB_10618573) were purchased from Gentex Inc (Irvine, CA, USA).

### 2.2. Cell Culture

Human neuroblastoma SH-SY5Y (RRID:CVCL_0019) cells were provided by the Korean Cell Line Bank (Seoul, Korea). SH-SY5Y cells were maintained in Minimum Essential Medium with Earle’s Balanced Salts solution (Hyclone, Logan, UT, USA, Cat# SH30024.01) supplemented with 10% heat-inactivated fetal bovine serum (Life Technologies Corporation, Carlsbad, CA, USA, Cat# 16000-044) and antibiotics at 37 °C in 5% CO_2_.

### 2.3. Gene Silencing

SH-SY5Y cells stably expressing shRNA targeting non-specific control or PPARδ (target sequence: 5′-CCGCAAACCCTTCAGTGATAT-3′) were generated by transducing lentiviral particles expressing each shRNA. For transduction, SH-SY5Y cells were seeded into 6-well plates as 1.6 × 10^5^ cells/well, cultured for 16 h, and subsequently transduced lentiviral particles with 8 μg/mL of hexadimethrine bromide (Sigma, St. Louis, MO, USA, Cat# H9268) in growth medium. The usage of lentiviral particles per well was the same number of seeded cell number (1 MOI). Transduced cells were selected by incubating the cells in culture medium containing 2 μg/mL puromycin for 7 days. *PPARD* silencing was verified by immunoblot analysis.

### 2.4. Cytotoxicity Assay

To assess cytotoxicity, 3-(4,5-dimethylthiazol-2-yl)-2,5-diphenyltetrazolium bromide (MTT, Sigma-Aldrich, Cat# M5655) and lactose dehydrogenase (LDH) release assays were conducted. For the MTT assay, SH-SY5Y cells were plated at 4 × 10^4^ cells/well in 24-well plates. Following incubation of the cells with the indicated reagents for 24 h, MTT solution was added to the culture medium and cells were incubated for an additional 2 h. After medium removal, formazan crystal formed in living cells was dissolved in acidified isopropanol. Absorbance was then measured at 570 nm using a Multiskan™ GO microplate spectrophotometer (Thermo Scientific, Waltham, MA, USA). To measure secreted LDH, culture media from cells treated with the specified reagents was collected. The level of released LDH was determined using a CytoTox 96 non-radioactive cytotoxicity assay kit (Promega, Madison, WI, USA, Cat# G1780). Absorbance was measured at 490 nm using a Multiskan™ GO microplate spectrophotometer (Thermo Scientific).

### 2.5. Measurement of Intracellular Iron Levels

Intracellular iron level was determined by two methods. First, a calcein fluorescent probe, which was inversely proportional to fluorescence intensity, was used to determine intercellular iron level as described previously [33]. Briefly, SH-SY5Y cells were plated at 1.6 × 10^5^ cells/well in 6-well plates (Corning Inc., Corning, NY, USA) and subsequently treated with the specified reagents. After incubation for the indicated duration, 500 nM calcein-AM (BD Biosciences, San Diego, CA, USA, Cat# 564061) was added to the culture medium. After treatment for 30 min at 37 °C, cells were then washed using phosphate-buffered saline (PBS) and green fluorescence was detected using an Eclipse Ti2 fluorescence microscope (Nikon, Minato, Tokyo, Japan). Second, a ferene-based colorimetric method was performed as described previously [34]. Labile iron concentrations were determined in cell lysates treated as above. Briefly, 100 μL of ammonium acetate buffer (2.5 M, pH 4.5) and 120 μL of labile iron working solution (5 mM ferene and 10 mM ascorbic acid prepared in 2.5 M ammonium acetate buffer, pH 4.5) were added to cell lysates. This mixture was vortexed and left overnight at room temperature. Then, the absorbance was measured at 595 nm in a Multiskan™ GO microplate spectrophotometer (Thermo Scientific). Iron concentrations were determined with a curve calibrated on iron standards and normalized to the amount of protein.

### 2.6. Western Blot Analysis

SH-SY5Y cells treated with the specified reagents for indicated durations were washed with ice-cold PBS and lysed in PRO-PREP™ Protein Extraction Solution (iNtRON Biotechnology, Seongnam, Korea, Cat# 17081). Cell lysate aliquots were subjected to sodium dodecyl sulfate-polyacrylamide gel electrophoresis (7.5% polyacrylamide) and transferred to Immobilon-P polyvinylidene difluoride membranes (Merck, Darmstadt, Germany, Cat# IPVH00010). Membranes were blocked for 2 h at ambient temperature with 5% non-fat milk suspended in Tris-buffered saline (TBS) containing 0.1% Tween-20 and subsequently incubated overnight at 4 °C with the indicated primary antibodies diluted in TBS containing 0.1% Tween-20. Membranes were subsequently incubated with a peroxidase-conjugated secondary antibody for 1 h at room temperature. After thorough washing of the membranes with TBS containing 0.1% Tween-20, chemiluminescence was visualized using WesternBright ECL (Advansta Inc., Menlo Park, CA, USA, Cat# K-12045-D50).

### 2.7. Real-Time PCR Analysis

Following treatment with each reagent for the indicated durations, total RNA was isolated using TRIzol reagent (Invitrogen, Carlsbad, CA, USA, Cat# 15596-018) and reverse-transcribed into cDNA using a TOPscript™ RT DryMIX kit (Enzynomics, Daejeon, Korea, Cat# RT201). Equal amounts of cDNA were diluted and amplified in a 20 μL reaction volume containing 1× PCR Master Mix (Solgent, Daejeon, Korea, Cat# SRH81-M40h) and random primers (0.5 μM). Real-time PCR was performed using a LightCycler^®^ 96 (Roche Diagnostics, Basel, Switzerland) with an initial denaturation for 15 min at 95 °C, followed by 40 cycles of 10 s at 95 °C, 10 s at 60 °C, and 30 s at 72 °C. The following primers were used: IRP1 (*ACO1*), 5′-GATTCAAGATATGGGCGCTTAC-3′ and 5′-TGCTGCGTGACATTCCAA-3′; *RPS18*, 5′- TGCGAGTACTCAACACCAAC-3′ and 5′-GTCTGCTTTCCTCAACACCA-3′; DMT1 (*SLC11A2*), 5′-GTTCTACTTGGGTTGGCAATGT-3′ and 5′-CCATAGAAACACACTGGCTCTGAT-3′; FPN1 (*SLC40A1*), 5′-CTACTTGGGGAGATCGGATGT-3′ and 5′-CTGGGCCACTTTAAGTCTAGC-3′; Ferritin heavy chain (*FTH1*), 5′-AACTACCACCAGGACTCAGA-3′ and 5′-ATCATCGCGGTCAAAGTAGT-3′; Ferritin light chain (*FTL*), 5′-ACCTCTCTCTGGGCTTCTAT-3′ and 5′-AGCTGGCTTCTTGATGTCCT-3′; TfR1 (*TFRC*), 5′-ACTTCTTCCGTGCTACTTCCAG-3′ and 5′-ACTCCACTCTCATGACACGATC-3′; and FSP1 (*AIFM2*), 5′- CTGAACGTCCCCTTCATGCT-3′ and 5′- ATCCCCACTACTAGCCCCTG-3′.

### 2.8. Measurement of Intracellular ROS

Intracellular ROS was measured by two methods. First, a H2DCF-DA fluorescent probe was used to determine ROS level as described previously [35]. Briefly, SH-SY5Y cells were plated at 1.6 × 10^5^ cells/well in 6-well plates (Corning Inc., Corning, NY, USA) and treated with the specified reagents. After incubation for the indicated duration, cells were treated with 50 μM 2′,7′-dichlorofluorescin diacetate (DCF-DA, Merck, Cat# 287810). Following incubation for 30 min at 37 °C, cells were washed with PBS and green fluorescence was then detected using a Ti2 fluorescence microscope (Nikon). Fluorescence intensity was quantified using Image J software (NIH, Bethesda, MD, USA). Second, an Amplex™ Red hydrogen peroxide assay kit was used to detect intracellular hydrogen peroxide according to the manufacturer’s instructions (Invitrogen, Carlsbad, CA, USA, Cat# A22188).

### 2.9. Measurement of Lipid Peroxidation

Lipid peroxidation was determined by two methods. First, a fluorescent probe BODIPY (581/591) (Invitrogen, Cat# D3861) was used to measure lipid peroxides as described previously [36]. Briefly, SH-SY5Y cells were plated at 1.6 × 10^5^ cells/well in 6-well plates (Corning) and treated with indicated reagents. After incubation for the indicated durations, cells were treated with 1 μM C11-BODIPY (581.591) for 30 min at 37 °C. C11-BODIPY fluorescence corresponding to oxidized or non-oxidized lipids was detected using an Eclipse Ti2 fluorescence microscope (Nikon). Fluorescence intensity was quantified using Image J software (NIH). Second, an EZ-lipid peroxidation (TBARS) assay kit was used to detect intracellular lipid peroxides according to the manufacturer’s instructions (DOGEN, Seoul, Korea, Cat# DG-TBA200).

### 2.10. Statistical Analysis

Data were analyzed using SigmaPlot 10 software (Systat, Chicago, IL, USA) and are expressed as means ± standard error (SE). Statistical significance was determined by one-way ANOVA with Tukey’s post hoc test or unpaired *t*-test with Welch’s correction using Prism 5 software (GraphPad Software, San Diego, CA, USA). *p* < 0.05 was considered statistically significant [37].

## 3. Results

### 3.1. GW501516 Activation of PPARδ Attenuates 6-OHDA-Triggered Cellular Damage in SH-SY5Y Cells

Because 6-OHDA induces degeneration of both noradrenergic and dopaminergic neurons and is known to contribute to PD pathology [4], we used 6-OHDA treatment as an in vitro model of PD. Cells were treated with various concentrations of 6-OHDA for 24 h. The lowest neurotoxic concentration was 20 μM 6-OHDA (Supplementary Figure S1). Accordingly, we used 20 μM 6-OHDA for subsequent experiments.

**Figure 1:**
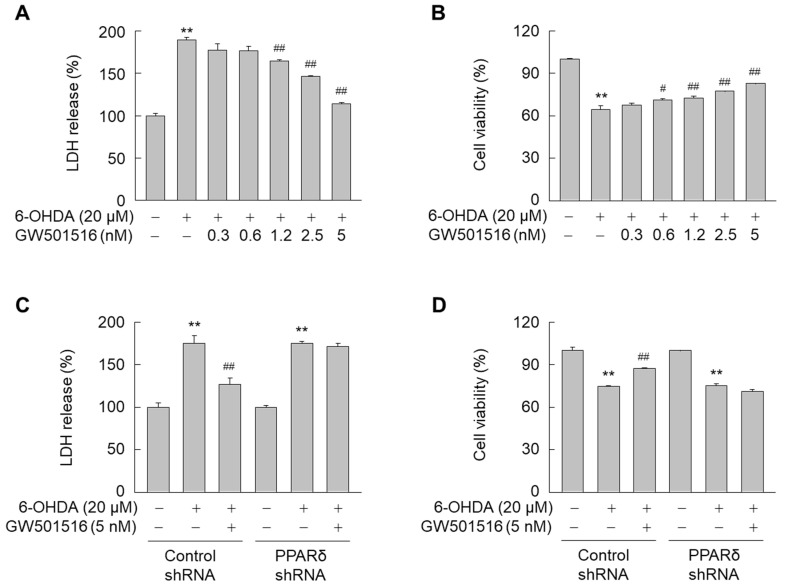
GW501516 PPARδ activation inhibits 6-OHDA-induced neurotoxicity in SH-SY5Y cells. (**A**,**B**) Cells pretreated with increasing concentrations of GW501516 for 8 h were exposed to 6-OHDA. After incubation for 24 h, LDH release (**A**) and MTT assays (**B**) were performed to evaluate 6-OHDA-induced cellular damage. (**C**,**D**) Cells stably expressing shRNA targeting scrambled sequences or PPARδ were pretreated with vehicle (DMSO) or GW501516 for 8 h and subsequently exposed to 6-OHDA. Following incubation for 24 h, cells were subjected to LDH release (**C**) and MTT assays (**D**). Results are expressed as means ± SE (*n* = 3). ** *p* < 0.01 relative to untreated group; ^#^ *p* < 0.05, ^##^ *p* < 0.01 relative to 6-OHDA-treated group.

Subsequently, we evaluated the effects of GW501516, a PPARδ-specific agonist against 6-OHDA neurotoxicity. When SH-SY5Y cells were treated with 6-OHDA for 24 h, LDH release was significantly increased. However, 6-OHDA-induced LDH release was dose-dependently decreased by GW501516 treatment (Figure 1A). Similarly, 6-OHDA-induced decrease in cell viability (MTT assay) was also reversed by GW501516 in a concentration-dependent manner (Figure 1B). 

To determine if the effects of GW501516 were PPARδ-dependent, the protective effects of GW501516 were evaluated in SH-SY5Y cells stably expressing shRNA targeting PPARδ or scrambled sequences. PPARδ protein levels were dramatically decreased in SH-SY5Y cells transduced with shRNA targeting PPARδ, but not scrambled sequence-targeting shRNA (Supplementary Figure S2). The neuroprotective effects of GW501516 against 6-OHDA were ablated by PPARδ-targeting shRNA, as demonstrated by both LDH release and MTT assay (Figure 1C,D). These results indicated that GW501516 attenuates 6-OHDA-induced neurotoxicity in a PPARδ-dependent manner.

### 3.2. GW501516 Activation of PPARδ Decreases 6-OHDA-Induced Iron Accumulation 

**Figure 2:**
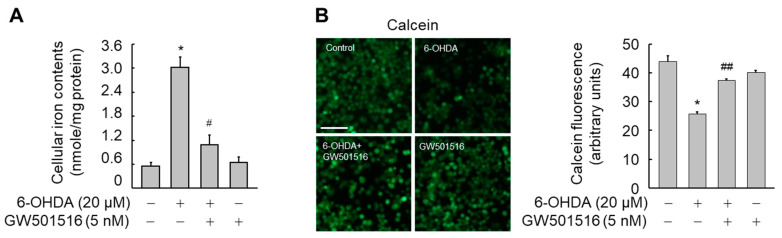
GW501516 activation of PPARδ suppresses 6-OHDA-triggered accumulation of intracellular iron. Cells pretreated with vehicle (DMSO) or GW501516 for 8 h were incubated with or without 6-OHDA for 24 h. (**A**) Ferene-based colorimetric method was used to determine the intracellular iron levels. (**B**) Intracellular iron levels were also determined by measuring the reduced fluorescence of calcein-AM under fluorescence microscopy and then calcein fluorescence was quantitated using cells treated with DMSO as control. Results are expressed as means ± SE (*n* = 3). Scale bar, 100 μm. * *p* < 0.05 relative to untreated group; ^#^ *p* < 0.05, ^##^ *p* < 0.01 relative to 6-OHDA-treated group.

Iron accumulation in dopaminergic neurons has been implicated in the pathogenesis of PD [15,16]. Moreover, 6-OHDA-induced reduction in viability is attenuated by ferroptosis inhibitors such as ferrostatin-1 and liproxstatin-1 (Supplementary Figure S3). Thus, we determined if the neuroprotective role of PPARδ in 6-OHDA-induced neurotoxicity was associated with the regulation of intracellular iron. When SH-SY5Y cells were exposed to 6-OHDA for 24 h, intracellular iron levels were markedly increased. However, 6-OHDA-induced iron accumulation was significantly decreased by GW501516 (Figure 2A,B).

**Figure 3:**
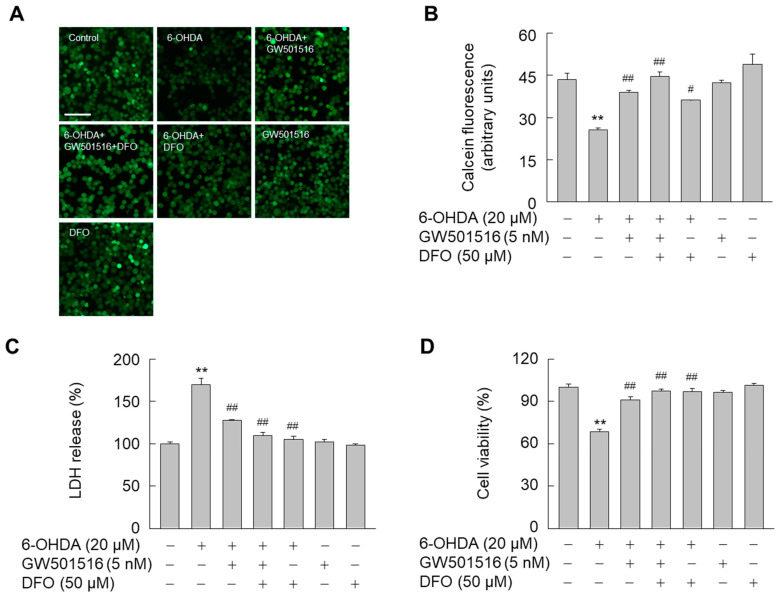
GW501516 activation of PPARδ suppresses 6-OHDA-triggered accumulation of intracellular iron. (**A**,**B**) Cells pretreated with vehicle (DMSO) or GW501516 for 8 h were treated with deferoxamine for 1 h and then incubated with or without 6-OHDA for 24 h. Intracellular iron levels were determined by measuring the reduced fluorescence of calcein-AM under fluorescence microscopy (**A**) and calcein fluorescence was quantitated using cells treated with DMSO as control (**B**). (**C**,**D**) In parallel experiments, 6-OHDA-induced neurotoxicity was evaluated by LDH release (**C**) and MTT assays (**D**). Results are expressed as means ± SE (*n* = 3). Scale bar, 100 μm. ** *p* < 0.01 relative to untreated group; ^#^ *p* < 0.05, ^##^ *p* < 0.01 relative to 6-OHDA-treated group.

To further examine the actions of PPARδ on the intracellular iron levels, we evaluated the effect of deferoxamine (DFO), an iron chelator. 6-OHDA-mediated increase in intracellular iron was also significantly reduced by DFO as did GW501516 (Figure 3A,B). Furthermore, DFO and GW501516 had similar protective effects against 6-OHDA-induced neuronal damage, as assessed by both LDH release (Figure 3C) and MTT assays (Figure 3D). Combined treatment of both GW501516 and DFO, however, did not have increased protective effects relative to GW501516 treatment alone in 6-OHDA-induced iron accumulation or neuronal damage. These results suggested that the neuroprotective effects of PPARδ against 6-OHDA are directly related to modulation of intracellular iron levels.

**Figure 4:**
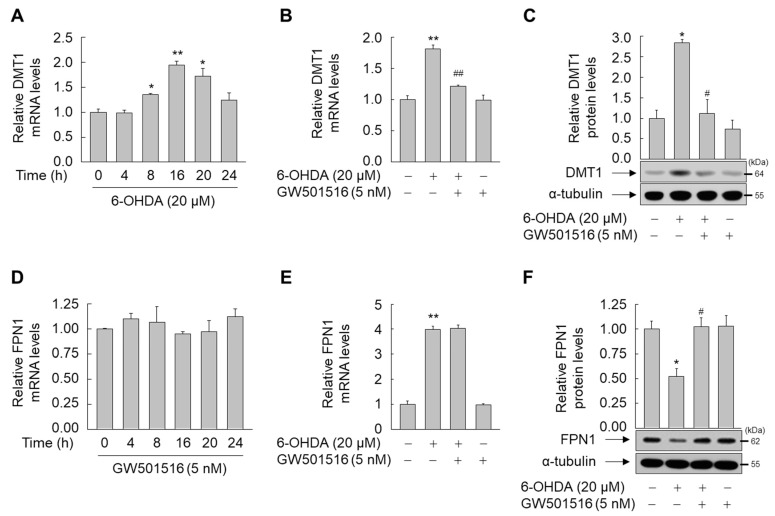
GW501516 activation of PPARδ regulates 6-OHDA-triggered expression of level of DMT1 and FPN1 protein, and DMT1 mRNA, but not FPN1 mRNA. (**A**) Cells were treated with 6-OHDA for the indicated durations. (**B**,**C**) Cells pretreated with DMSO or GW501516 for 8 h were incubated with or without 6-ODHA for 16 h. (**D**) Cells were treated with GW501516 for the indicated durations. (**E**,**F**) Cells pretreated with DMSO or GW501516 for 8 h were incubated with or without 6-ODHA for 16 h. Total RNA and protein were extracted, and mRNA and protein levels were analyzed by real-time PCR (**A**,**B**,**D**,**E**) and Western blot (**C**,**F**), respectively. RPS18 and α-tubulin were used as internal controls for real-time PCR and Western blot, respectively. Results are expressed as means ± SE (*n* = 3). An image analyzer was used to quantify band intensity of Western blot, and the ratio of protein to α-tubulin is indicated above each lane. * *p* < 0.05, ** *p* < 0.01 relative to the untreated group; ^#^ *p* < 0.05, ^##^ *p* < 0.01 relative to the 6-OHDA-treated group.

To assess the regulatory mechanisms of PPARδ-mediated iron homeostasis, we examined the effects of GW501516 on the expression of key regulators of iron homeostasis, DMT1 and FPN1. Treatment of SH-SY5Y cells with 6-OHDA time-dependently increased mRNA levels of DMT1, an iron importer, which was prevented by pre-treatment with GW501516 (Figure 4A,B). Similarly, 6-OHDA increased DMT1 protein levels, which was reduced by GW501516 pre-treatment (Figure 4C).

Subsequently, we evaluated the effects of 6-OHDA and GW501516 on the expression of FPN1, an iron exporter. Time-dependent exposure of SH-SY5Y cells to GW501516 did not affect FPN1 mRNA levels (Figure 4D). Although 6-OHDA increased FPN1 mRNA in SH-SY5Y cells, GW501516 co-treatment did not reverse this effect (Figure 4E). Contrastingly, 6-OHDA decreased protein levels of FPN1 in SH-SY5Y cells, which was prevented by GW501516 pre-treatment, implying that PPARδ modulates FPN1 protein levels, but not mRNA levels, potentially by regulating FPN1 translation (Figure 4F).

**Figure 5:**
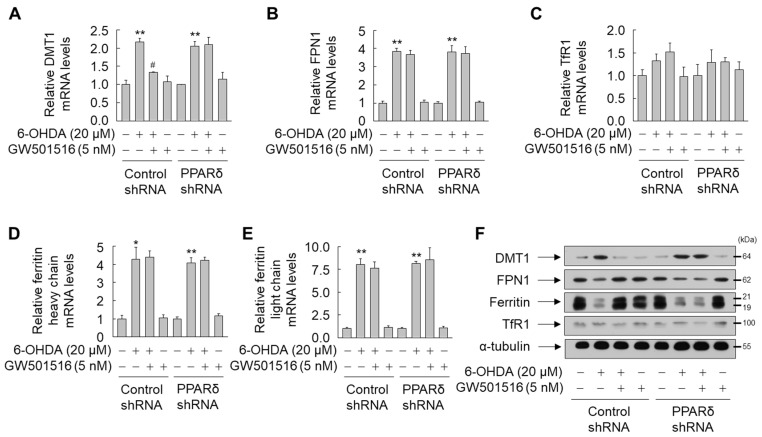
PPARδ knockdown abrogated the effects of GW501516 on 6-OHDA-triggered protein levels of DMT1, FPN1, and Ferritin, and mRNA expression of DMT1. Cells stably expressing shRNA targeting scrambled sequences or PPARδ were pretreated with DMSO or GW501516 for 8 h, and subsequently incubated with or without 6-OHDA for 16 h. Total RNA and protein were extracted, and levels of mRNA and protein were analyzed by real-time PCR (**A**–**E**) and Western blot (**F**). RPS18 and α-tubulin were used as internal controls for real-time PCR and Western blot, respectively. Results are expressed as means of triplicate ± SE (**A**–**D**). * *p* < 0.05, ** *p* < 0.01 relative to untreated group; ^#^ *p* < 0.05 relative to 6-OHDA-treated group.

To directly assess the role of PPARδ in GW501516-mediated expression of key iron-regulatory proteins including DMT1, FPN1, ferritin, and TfR1, the effect of GW501516 was examined in SH-SY5Y cells stably expressing shRNA targeting PPARδ or scrambled sequences. GW501516 suppression of DMT1 mRNA and protein levels in 6-OHDA-challenged cells was significantly attenuated in SH-SY5Y cells stably expressing PPARδ-targeting shRNA, but not control shRNA (Figure 5A,F). Contrastingly, GW501516 did not alter mRNA levels of FPN1 and ferritin in 6-OHDA-treated cells stably expressing shRNA targeting either PPARδ or scrambled sequences (Figure 5B,D,E). However, GW501516 reversed the 6-OHDA-triggered decrease in FPN1 and ferritin protein in SH-SY5Y cells expressing scrambled shRNA, but not in cells stably expressing PPARδ-targeting shRNA, indicating that PPARδ regulates translation of FPN1 and ferritin rather than transcription (Figure 5F). The expression of TfR1 mRNA and protein was not significantly affected by either 6-OHDA or GW501516 (Figure 5C,F).

### 3.3. GW501516 Activation of PPARδ Suppresses IRP1 Expression in SH-SY5Y Cells

**Figure 6:**
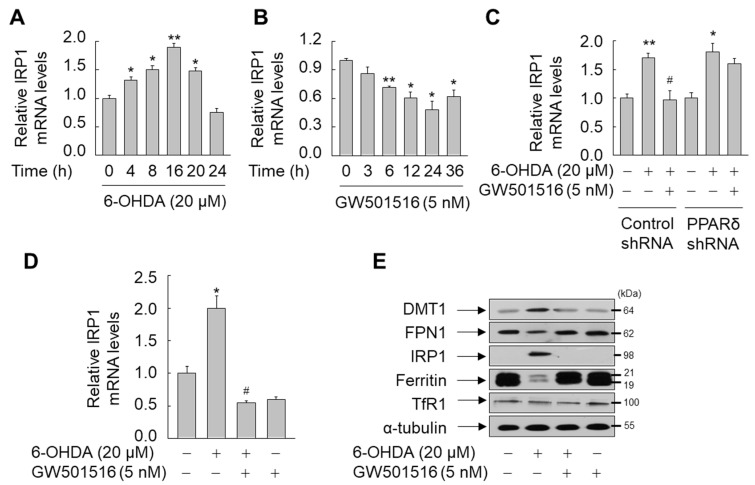
GW501516 PPARδ activation suppresses 6-OHDA-induced expression of IRP1 in SH-SY5Y cells. (**A**,**B**) Cells were treated with 6-OHDA (**A**) or GW501516 (**B**) for the indicated durations. (**C**) Cells stably expressing shRNA targeting scrambled sequences or PPARδ were pretreated with DMSO or GW501516 for 8 h and subsequently incubated in the presence or absence of 6-ODHA for 16 h. (**D**,**E**) Cells pretreated with DMSO or GW501516 for 8 h were incubated with or without 6-ODHA for 16 h. Total RNA and protein were extracted, and levels of mRNA and protein were analyzed by real-time PCR (**A**–**D**) and Western blot (**E**). The immunoblots are separate from those shown in Figure 4C,F. RPS18 and α-tubulin were used as internal controls for real-time PCR and Western blot, respectively. Results are expressed as means of triplicate ± SE (**A**–**D**). * *p* < 0.05, ** *p* < 0.01 relative to untreated group; ^#^ *p* < 0.05 relative to 6-OHDA-treated group.

To further elucidate the molecular mechanism of PPARδ-mediated modulation of key iron-regulatory proteins in SH-SY5Y cells, we determined the effect of GW501516 on the expression of IRP1, which is known to regulate the expression of these key proteins by binding IREs in the 3′ or 5′ UTRs of these genes [38]. When SH-SY5Y cells were exposed to 6-OHDA, IRP1 mRNA was increased, reaching a maximum of two-fold upregulation after 16 h and declining to basal levels after 24 h (Figure 6A). Contrastingly, GW501516 monotreatment downregulated IRP1 mRNA in a time-dependent manner (Figure 6B). Consistent with these results, shRNA-targeted knockdown of PPARδ suppressed the effects of GW501516 on 6-OHDA-induced IRP1 upregulation (Figure 6C). Consistently, the pre-treatment with GW501516 attenuated 6-OHDA-induced upregulation of IRP1 mRNA (Figure 6D). This PPARδ-mediated downregulation of IRP1 was correlated with expression of target proteins, in which GW501516 markedly reversed the effects of 6-OHDA on the protein expression of DMT1, FPN1, and ferritin, but not TfR1 (Figure 6E). In contrast, GW501516 did not alter markedly the mRNA and protein expression of IRP2 (Supplementary Figure S4), which has high sequence homology and similar biochemical activities to that of IRP1 [39]. This result may explain the effect of GW501516 on the expression of TfR1, which is more affected by IRP2 than IRP1 [40]. Additionally, GPX4 or FSP1, important effectors in anti-ferroptotic signaling [41,42], were not regulated in presence of GW501516 (Supplementary Figure S5).

### 3.4. GW501516 Activation of PPARδ Decreases 6-OHDA-Triggered Accumulation of ROS and Lipid Peroxides

**Figure 7:**
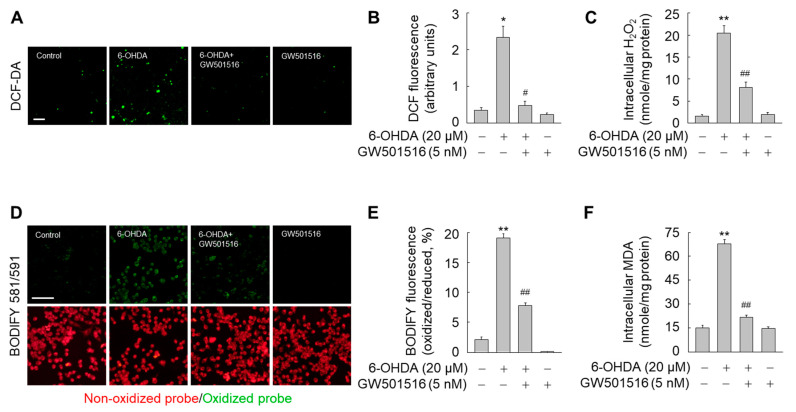
GW501516 PPARδ activation suppresses 6-OHDA-triggered accumulation of ROS and lipid peroxides. (**A**–**C**) Cells pretreated with DMSO or GW501516 for 8 h were incubated with or without 6-ODHA for 24 h. Intracellular ROS levels were determined using DCF-DA dye under fluorescence microscopy (**A**) and the fluorescence was quantitated using cells treated with DMSO as control (**B**). The levels of hydrogen peroxide were detected using Amplex™ Red hydrogen peroxide assay kit (**C**). (**D**–**F**) In parallel experiments, lipid peroxides were determined using C11-BODIPY 581/591 under fluorescence microscopy (**D**) and BOFIFY fluorescence was quantitated using cells treated with DMSO as control (**E**). Intracellular malondialdehyde (MDA) levels were determined using EZ-lipid peroxidation (TBARS) assay kit (**F**). Results are expressed as means ± SE (*n* = 3). Scale bar, 100 μm. * *p* < 0.05, ** *p* < 0.01 relative to untreated group; ^#^ *p* < 0.05, ^##^ *p* < 0.01 relative to 6-OHDA-treated group.

**Figure 8:**
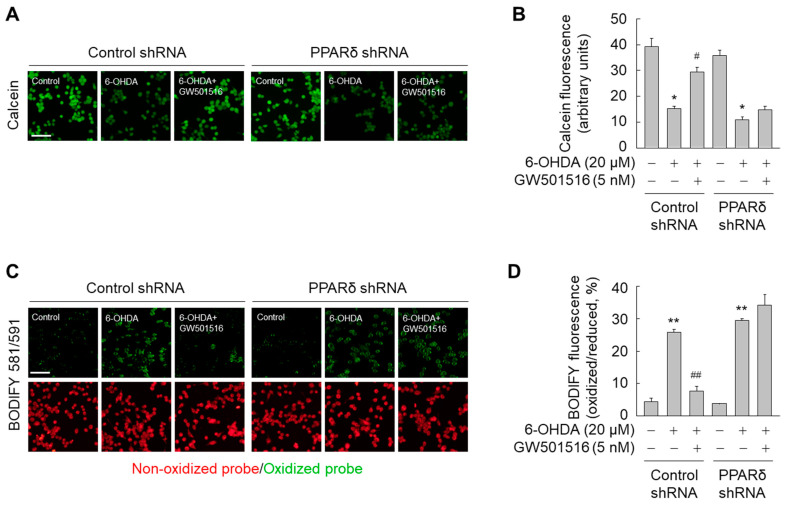
PPARδ knockdown abrogates the effects of GW501516 on intracellular iron accumulation and lipid peroxidation in SH-SY5Y cells. (**A**,**C**) Cells stably expressing shRNA targeting scrambled sequences or PPARδ were pretreated with DMSO or GW501516 for 8 h, and subsequently incubated with or without 6-OHDA for 24 h. Cells were then incubated in medium containing calcein-AM (**A**) or C11-BODIPY 581/591 (**C**) to detect intracellular iron and lipid peroxides, respectively. (**B**,**D**) Fluorescence signals corresponding to intracellular iron (**B**) and oxidized C11-BODIPY (**B**) were detected using fluorescence microscopy and fluorescence intensities were quantified. Results are expressed as means ± SE (*n* = 3). Scale bar, 100 μm. * *p* < 0.05, ** *p* < 0.01 relative to untreated group; ^#^ *p* < 0.05, ^##^ *p* < 0.01 relative to 6-OHDA-treated group.

Because pathologically increased intracellular iron levels trigger generation of ROS and lipid peroxides [7], we determined if 6-OHDA affected intracellular ROS and lipid peroxides. Exposure of SH-SY5Y cells to 6-OHDA for 24 h dramatically increased the levels of intracellular ROS and hydrogen peroxide (Figure 7A–C). Consistent with increased intracellular ROS levels, lipid peroxides were significantly increased in 6-OHDA-treated cells (Figure 7D–F). However, 6-OHDA-triggered accumulation of intracellular ROS and lipid peroxides was almost completely abolished by GW501516 pre-treatment (Figure 7). Furthermore, the beneficial effects of GW501516 were ablated in SH-SY5Y cells stably expressing PPARδ-targeting shRNA, but not control shRNA (Figure 8). These results indicated that PPARδ elicits its neuroprotective effects against 6-OHDA-induced toxicity in part through ferroptotic signaling.

## 4. Discussion

Among the PPAR subtypes, PPARδ is most abundantly expressed in the brain, suggesting its importance in neuronal function [43]. Although the anti-inflammatory and metabolic roles of PPARδ have been extensively studied [22], the potential protective roles of PPARδ against neuronal cell death in the dopaminergic system, including against ferroptosis, have not been evaluated. The present study demonstrated that activation of PPARδ with a synthetic PPARδ-specific ligand, GW501516, attenuates 6-OHDA-induced neuronal damage, a major pathological event in PD, by suppressing intracellular iron accumulation and lipid peroxidation, the primary phenotypes of ferroptosis. The anti-ferroptotic effects of PPARδ were associated with the key iron regulatory protein IRP1, which functions as a translational regulator of the cellular iron transporters DMT1 and FPN1. 

The anti-ferroptotic effects of PPARδ are related directly to blockade of neuronal damage induced by 6-OHDA. A previous study demonstrated that pathologically increased intracellular iron levels are a critical initiator of ferroptosis, and that multiple iron metabolism-associated genes such as *HAMP*, *FTH1*, *HSPB1*, *TF*, *SLC40A1*, *TFRC,* and *STEAP3*, are involved in this event [10,44]. In addition, iron chelation reversed ferroptotic cell death triggered by the ferroptosis inducers erastin, sulfasalazine, and RSL3, suggesting a critical role for iron accumulation in ferroptotic cell death [7]. Consistent with previous studies, activation of PPARδ by the specific ligand GW501516 attenuated 6-OHDA-induced intracellular iron accumulation. This effect of PPARδ was mediated in part by modulation of the key iron-regulatory proteins DMT1, FPN1, and ferritin. Our findings suggest that PPARδ affects DMT1, FPN1, and ferritin, but not TfR1, by downregulating IRP1, which modulates expression of these iron transporters. 

Although calcium-independent phospholipase A2 beta and protein kinase C are implicated in regulation of IRP1 expression [45,46], 6-OHDA treatment did not affect either of these pathways in SH-SY5Y cells (data not shown). The molecular mechanisms underlying PPARδ-mediated downregulation of IRP1 are unclear. However, the present findings demonstrated that activation of PPARδ by GW501516 dramatically attenuated 6-OHDA-induced intracellular iron accumulation by regulating iron transporter expression. Accordingly, further studies will determine the precise molecular mechanism for PPARδ-mediated downregulation of IRP1.

Although the precise molecular mechanisms of PPARδ-mediated neuroprotection from 6-OHDA are lesser known than its roles in cellular inflammation and metabolism [22], the present observations are consistent with a previous report demonstrating that PPARδ improves motor impairment and dopaminergic neurodegeneration by inhibiting neuroinflammation in the MPTP mouse model of PD [28]. In addition, the PPARδ agonists GW501516 and L-165041 significantly suppressed MPTP-triggered depletion of striatal dopamine in the murine brain [27]. The other PPARδ agonist GW0742 also improved MPTP-induced cognitive impairment by suppressing oxidative damage and DNA fragmentation in a rat model of PD [47]. Although the function of the PPARδ in the pathogenesis of PD is not fully elucidated, the present findings clearly indicate that activation of PPARδ with GW501516 attenuates 6-OHDA-induced neuronal damage in SH-SY5Y neuroblastoma cells, indicating the potential therapeutic potential of PPARδ as a novel approach to treatment for neurodegenerative diseases, particularly PD. 

Ferroptosis is a potential mechanism for degeneration of dopaminergic neurons in the context of PD. Excess iron accumulation is present in the substantia nigra of autosomal recessive juvenile parkinsonism patients, suggesting that regulation of iron levels and iron-mediated oxidative stress could suppress parkinsonism [16]. Consistent with this notion, oxidated cholesterol metabolites are elevated in a dopaminergic cell line, suggesting that oxidative stress exacerbates neurodegeneration via generation of cholesterol aldehydes [48]. In fact, iron-dependent cell death, termed ferroptosis, includes induction of oxidative stress and is implicated in the pathogenesis of PD [7,49]. Linkage between ferroptosis and neuronal atrophy in dopaminergic and noradrenergic neurons has been mainstream opinion since it was discovered [50,51]. Moreover, ferritinophagy accompanying ferroptosis is a form of autophage involving iron-mediated ferroptosis, and blockade of ferritinophagy may be an important strategy for blocking ferroptosis-mediated cell death [52]. In this context, our result indicates that the PPARδ might associated with ferrototic cells death by regulating the expression of IRP1, which is known as a ferroptotic effector that acts by modulating the level of iron homeostasis-associated proteins such as DMT1, FPN1 and Ferritin.

Multiple antioxidants such as coenzyme Q10, vitamin E, and creatine were ineffective in clinical trials for PD [53,54,55]. However, the iron chelator deferiprone improved motor function while raising striatal dopamine by reducing labile iron levels and oxidative stress-induced damages in mice [56]. Similar improvements in substantia nigra iron deposits and motor function were also observed in a pilot clinical trial for PD patients [56].

## 5. Conclusions

Consistent with these previous studies, we demonstrated in the present report that activation of PPARδ by GW501516 attenuated 6-OHDA-triggered intracellular iron accumulation by regulating the expression of iron transport proteins. PPARδ modulation of intracellular iron levels was accompanied by decreased ROS and lipid peroxide levels in 6-OHDA-treated cells pretreated with GW501516. These observations provide insight into the potential protective and anti-ferroptotic roles of PPARδ in the context of PD.

## Data Availability

Data are contained within the article or Supplementary Materials.

## Supplementary Materials

The following supporting information can be downloaded at: https://www.mdpi.com/article/10.3390/antiox11050810/s1. Figure S1: Effects of 6-OHDA on SH-SY5Y cell viability. Figure S2: Effects of siRNA targeting PPARδ or scrambled sequences on expression of PPARδ protein in SH-SY5Y cells. Figure S3: Effects of Ferrostatin-1, Liproxstatin-1, and α-tocopherol on 6-OHDA-induced neurotoxicity of SH-SY5Y cells. Figure S4: Effects of GW501516 on the level of *IRP2* expression in SH-SY5Y cells treated with or without 6-OHDA. Figure S5: Effects of GW501516 on the levels of GPX4 and AIFM2 in SH-SY5Y cells treated with or without 6-OHDA.
