# Supplementary material for: PPARδ Activation Mitigates 6-OHDA-Induced Neuronal Damage by Regulating Intracellular Iron Levels
Source: Antioxidants (Basel). 2022 Apr 21;11(5):810. doi: 10.3390/antiox11050810 (PMC9137940; doi:10.3390/antiox11050810)
Supplement: Supplementary file 1 [file antioxidants-11-00810-s001.zip › antioxidants-1667050-supplementary.pdf]

Supplementary figures

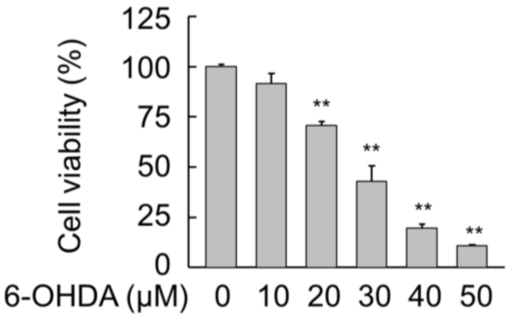

Figure S1. Effects of 6-OHDA on SH-SY5Y cell viability. Cells were treated with increasing doses of 6-OHDA for 24 h and then subjected to an MTT assay to determine cell viability. A significant and dose-dependent decrease in cell viability was observed in 6-OHDA-treated SH-SY5Y cells. Data are obtained from three independent biological replicates (n=3) and plotted. $^{**}p < 0.01$ relative to untreated group.

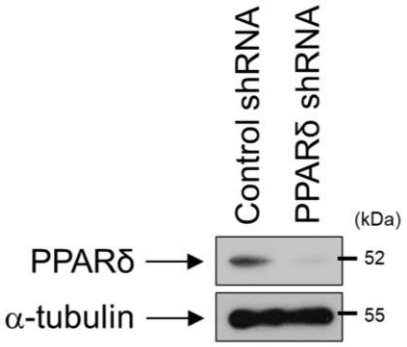

Figure S2. Effects of siRNA targeting PPARδ or scrambled sequences on expression of PPARδ protein in SH-SY5Y cells. Cells were transduced with lentiviral particles expressing each shRNA in growth medium. After selection using puromycin for 7 days, PPARδ expression was evaluated by immunoblot. PPARδ expression was markedly reduced in the presence of shRNA targeting PPARδ relative to control.

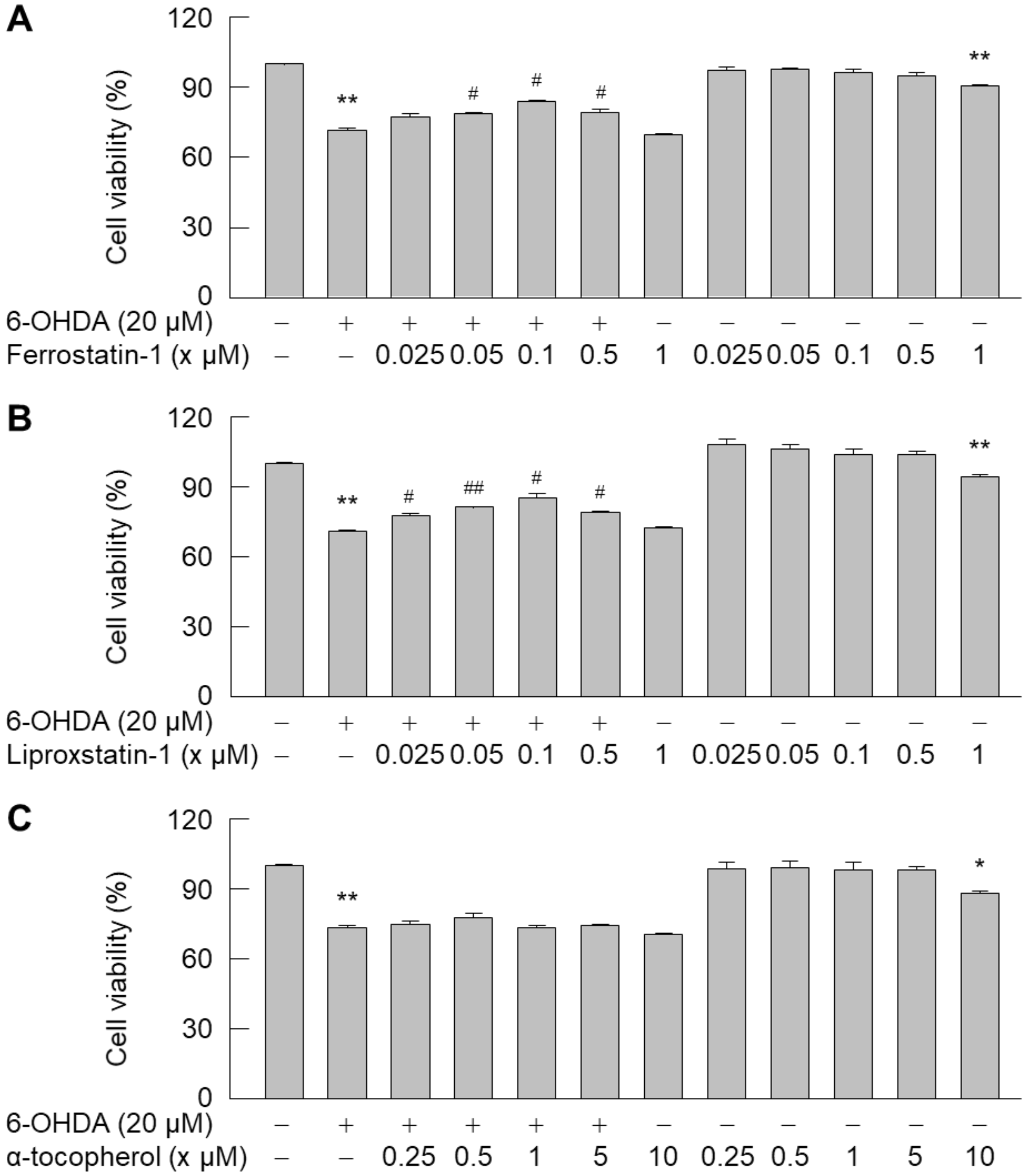

Figure S3. Effects of Ferrostatin-1, Liproxstatin-1, and α-tocopherol (Vitamin E) on 6-OHDA-induced neurotoxicity of SH-SY5Y cells. Cells pretreated with Ferrostatin-1, Liproxstatin-1, or alpha-tocopherol (Vitamin E) for 2 h were incubated with 6-OHDA for 24 h and then subjected to an MTT assay to determine cell viability. Data are obtained from three independent biological replicates (n=3) and plotted. $^{*}p < 0.05$, $^{**}p < 0.01$ relative to untreated group; $^{\#}p < 0.05$, $^{\#\#}p < 0.01$ relative to 6-OHDA-treated group.

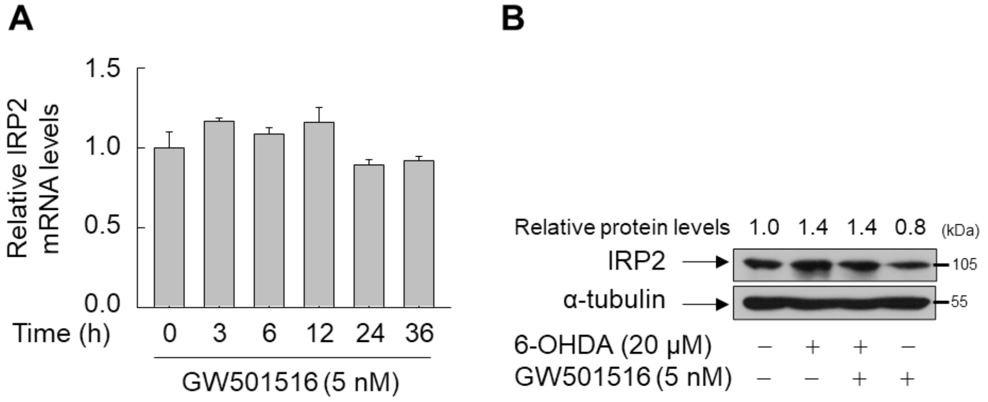

Figure S4. Effects of GW501516 on the level of *IRP2* expression in SH-SY5Y cells treated with or without 6-OHDA. (A) Cells were treated with GW501516 for the indicated time periods. (B) Cells pretreated with DMSO or GW501516 for 8 h were incubated with or without 6-ODHA for 16 h. Total RNA and protein were extracted, and the levels of mRNA and protein were analyzed by real-time PCR (A) and Western blot (B), respectively. *RPS18* and α-tubulin were used as internal controls for real-time PCR and Western blot, respectively. Results are expressed as the means ± SE (n = 3). An image analyzer was used to quantify the band intensity of Western blot, and the ratio of protein to α-tubulin is indicated above each lane.

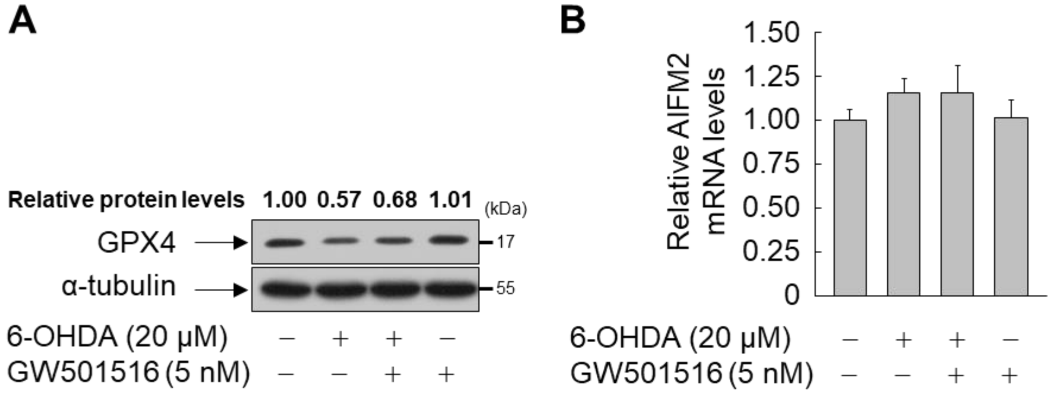

Figure S5. Effects of GW501516 on the levels of GPX4 and AIFM2 in SH-SY5Y cells treated with or without 6-OHDA. (A) Cells seeded in 60 mm culture dishes were pretreated

with DMSO or GW501516 for 8 h and then incubated with 6-OHDA for 16 h. Total protein and RNA were extracted, and the levels of protein and mRNA were analyzed by Western blot (A) and real-time PCR (B), respectively. α-tubulin and *RPS18* were used as internal controls for Western blot and real-time PCR, respectively. Results are expressed as the means ± SE (n = 3). An image analyzer was used to quantify band intensity of Western blot, and the ratio of protein to α-tubulin is indicated above each lane.
